# IL‐18‐induced expression of high‐affinity IL‐2R on murine NK cells is essential for NK‐cell IFN‐γ production during murine *Plasmodium yoelii* infection

**DOI:** 10.1002/eji.201546018

**Published:** 2015-10-21

**Authors:** Kerstin A. Stegmann, J. Brian De Souza, Eleanor M. Riley

**Affiliations:** ^1^ Faculty of Infectious and Tropical DiseasesDepartment of Immunology and InfectionLondon School of Hygiene and Tropical MedicineLondonUK; ^2^ Division of Infection and ImmunityUniversity College LondonLondonUK

**Keywords:** CD25, IL‐12, IL‐18, Malaria, NK cells

## Abstract

Early production of pro‐inflammatory cytokines, including IFN‐γ, is essential for control of blood‐stage malaria infections. We have shown that IFN‐γ production can be induced among human natural killer (NK) cells by coculture with *Plasmodium falciparum* infected erythrocytes, but the importance of this response is unclear. To further explore the role of NK cells during malaria infection, we have characterized the NK‐cell response of C57BL/6 mice during lethal (*Py*YM) or nonlethal (*Py*17XNL) *P. yoelii* infection. Ex vivo flow cytometry revealed that NK cells are activated within 24 h of *Py*17XNL blood‐stage infection, expressing CD25 and producing IFN‐γ; this response was blunted and delayed during *Py*YM infection. CD25 expression and IFN‐γ production were highly correlated, suggesting a causal relationship between the two responses. Subsequent in vitro experiments revealed that IL‐18 signaling is essential for induction of CD25 and synergizes with IL‐12 to enhance CD25 expression on splenic NK cells. In accordance with this, *Py*17XNL‐infected erythrocytes induced NK‐cell CD25 expression and IFN‐γ production in a manner that is completely IL‐18‐ and partially IL‐12‐dependent, and IFN‐γ production is enhanced by IL‐2. These data suggest that IL‐2 signaling via CD25 amplifies IL‐18‐ and IL‐12‐mediated NK‐cell activation during malaria infection.

## Introduction

Malaria is a major global health problem causing in excess of 600,000 deaths per year, 80% of these in sub‐Saharan Africa 1. Human immunoepidemiological studies as well as murine models of *Plasmodium* spp. infection have proven valuable in elucidating both innate and adaptive responses to malaria and their contribution to protective immunity 2, 3. Collectively, these studies suggest an important role for the cytokine IFN‐γ in clearance of blood‐stage infections. In particular, a robust IFN‐γ response in the first 24–48 h after blood‐stage infection correlates with a favorable outcome and long‐term survival in mouse models 4, 5. Although a number of immune cells have been reported to produce IFN‐γ, T lymphocytes and natural killer (NK) cells are by far the most proficient producers of this cytokine 6 suggesting that they may be key players in protective immunity to malaria.

Freshly isolated human NK cells can produce large amounts of IFN‐γ within 12–18 h of coculture with *Plasmodium falciparum* infected red blood cells (iRBCs) 7, 8. NK‐cell activation depends upon cytokine (IL‐12 and IL‐18) and contact‐dependent signals from monocytes and myeloid DCs 9 and is markedly amplified by IL‐2 10. Importantly, recent evidence from a humanized mouse model indicates that human NK cells can eliminate *P. falciparum* iRBC 11. The role of NK cells during murine blood‐stage malaria infections is however disputed and their mode of activation is less well studied, although there is a clear role for IL‐12 2. Proliferation and expansion of the peripheral blood NK‐cell population, together with upregulation of interferon associated gene transcripts, occurs within the first 24 h of *Plasmodium chabaudi* infection 12 and NK depletion with anti‐asialo GM1 antibodies leads to higher parasitemia, reduced DC activation, and reduced CD4^+^ T‐cell priming 13, 14. However, NK‐cell depletion with anti‐NK1.1 antibodies reportedly either increased mortality 15 or had no effect on the course of *P. chabaudi* infection 16. In *Plasmodium berghei* XAT infections, NK‐cell lytic activity is increased but NK depletion with anti‐NK1.1 antibodies does not affect parasite clearance 17. In nonlethal *Plasmodium yoelii* infections, NK cells have been shown to contribute to liver‐stage immunity 18, 19 and to be activated and secrete IFN‐γ during the first 24 h of blood‐stage infection 5, 20 but their contribution to protection is disputed; much less in the way of NK activation is observed during the early stage of lethal *P. yoelii* infections 20. Some of this confusion may arise from the lack, until recently, of highly specific reagents for identification and depletion of murine NK cells: both anti‐CD49b (DX5) and anti‐NK1.1 mark and delete subsets of T cells as well as NK cells. However, the identification of NKp46 as a highly specific NK‐cell marker 21 is allowing a more precise analysis of their role during malaria and other infections.

Here, we have investigated the very early NK‐cell response to two closely related strains of the rodent malaria parasite, *P. yoelii* (*Py*) *Py*17XNL causes a self‐resolving infection whereas *Py*YM replicates very rapidly, killing infected mice within 1 week. We find that NK cells are more quickly and more extensively activated during nonlethal *Py*17XNL infection than during lethal *Py*YM infection, that this correlates with more rapid and more extensive upregulation of the IL‐2 receptor α‐subunit (CD25) and thus with expression of high‐affinity IL‐2 receptor, and that expression of CD25 and secretion of IFN‐γ by NK cells is partially dependent upon IL‐12, is augmented by IL‐2, and totally dependent upon signaling through the IL‐18 receptor.

## Results

### NK cells upregulate the high‐affinity IL‐2R and secrete IFN‐γ within 24 h of *P. yoelii* infection

**Figure 1:**
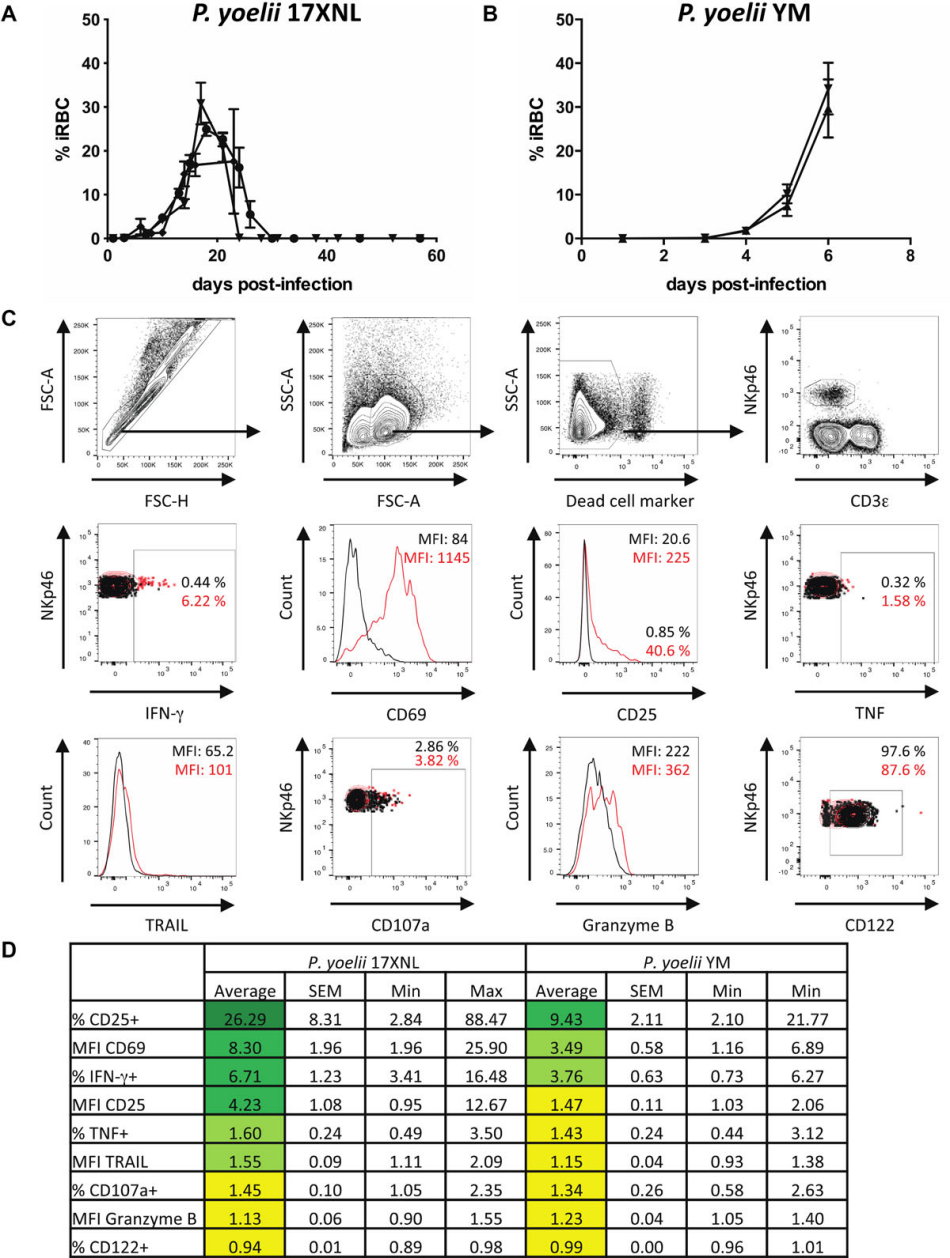
Early NK‐cell activation during lethal and nonlethal *P. yoelii* infection. C57BL/6 mice were infected i.p. with 10^5^ RBCs infected with (A) *P. yoelii* 17XNL or (B) *P. yoelii* YM. Each line represents the mean (SEM) parasitemia for groups of three to five mice in each of three (A) or two (B) independent experiments. (C) Splenic NK cells were identified by flow cytometry. Lymphocytes were gated after exclusion of cell aggregates, followed by exclusion of dead cells. NK cells were identified as NKp46^+^ CD3ε^–^ lymphocytes. Overlays are shown for activation and functional markers. Black = naive animal; red = day 1 of *P. yoelii* 17XNL infection. (D) Changes (fold increase) in frequency (%) or expression levels (MFI) of activation and functional markers on murine splenic NK cells 24 h after infection with *P. yoelii* 17XNL or *P. yoelii* YM compared to naive controls were determined by flow cytometry. Data are shown as mean, SEM, and range for *P. yoelii* 17XNL (*n* = 13 mice) and *P. yoelii* YM (*n* = 10 mice) infection pooled from three (*Py*17XNL) or two (*Py*YM) independent experiments.

In line with previous studies 20, iRBC became visible by microscopy approximately 5 days postinfection (p.i.) with 10^5^ nonlethal *Py*17XNL iRBC (Fig. 1A); parasitemia increased to a peak of approximately 30% from 17 to 21 days p.i. and was cleared by 28 days p.i. By contrast, during lethal *Py*YM infection, iRBC became visible by day 3 p.i. and parasitemia reached approximately 35% by 6 days p.i. (Fig. 1B) when mice were euthanized (according to welfare guidelines).

Splenic NK‐cell responses were analyzed directly ex vivo by flow cytometry. After exclusion of cell aggregates and dead cells, NK cells were identified as CD3^−^ and NKp46^+^ lymphocytes and examined for expression of functional markers (Fig. 1C). In line with previous studies 4, 5, 22, IFN‐γ was induced in NK cells within 24 h of nonlethal *Py*17XNL infection; CD69 and, somewhat surprisingly, CD25 were also induced on NK cells. TNF, TRAIL, and CD107a were modestly upregulated but there was little or no change in Granzyme B or CD122 expression (Fig. 1D). NK cells were also activated—although to a lesser extent—during the first 24 h of lethal *Py*YM infection (Fig. 1D) with IFN‐γ production, CD69 and CD25 expression being upregulated in some but not all animals.

**Figure 2:**
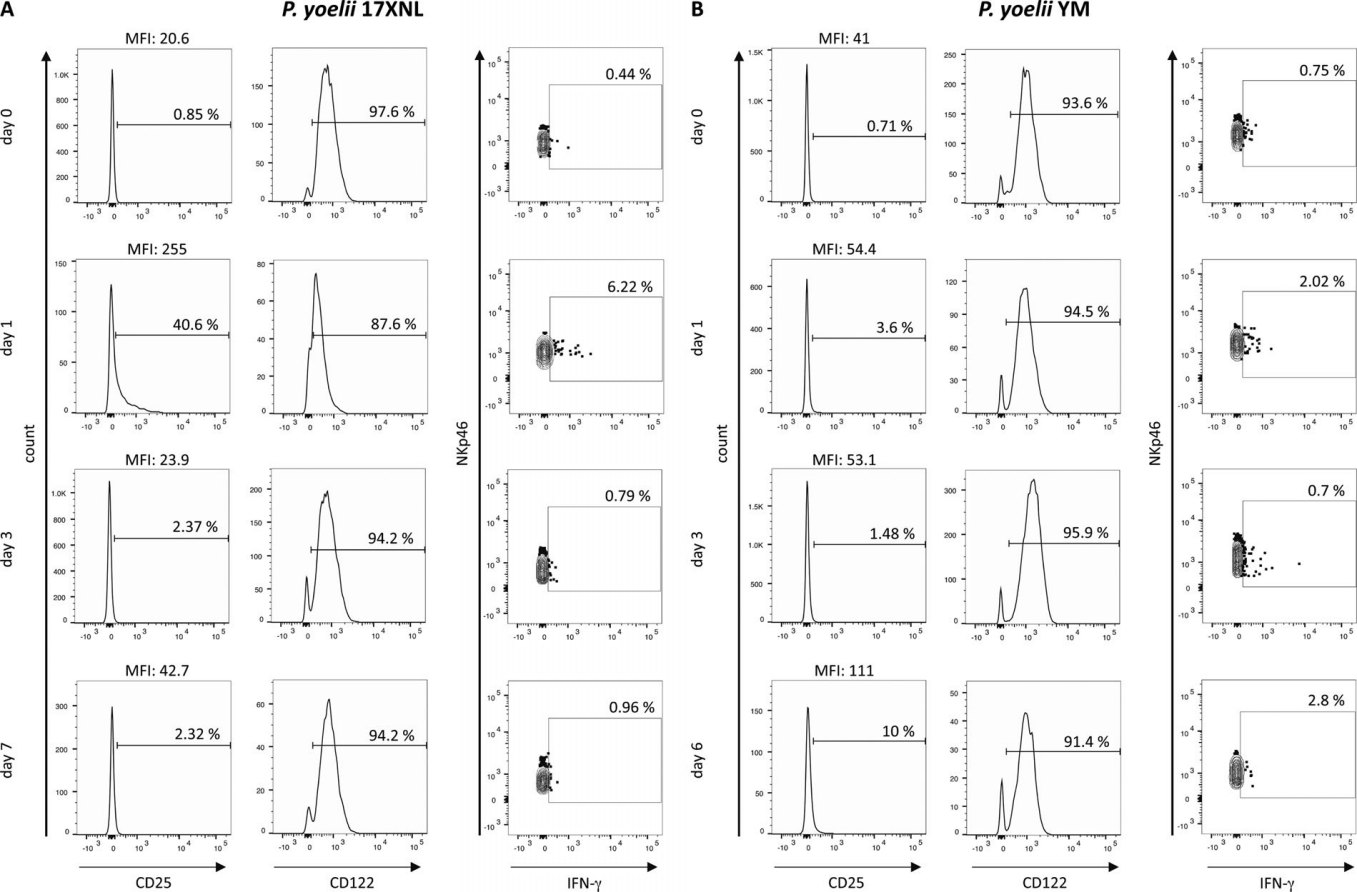
Expression of CD25 and IFN‐γ by splenic NK cells during nonlethal *P. yoelii* infection. Representative flow cytometry histograms of CD122 and CD25 expression and dot plots of IFN‐γ expression by NK cells (NKp46^+^CD3ε^−^) on days 0, 1, 3, and 6 or 7 after infection with (A) *P. yoelii* 17XNL or (B) *P. yoelii* YM. The gating strategy was as shown in Figure 1C. The percentages of positive cells and the MFI of the entire NKp46^+^ population are shown.

**Figure 3:**
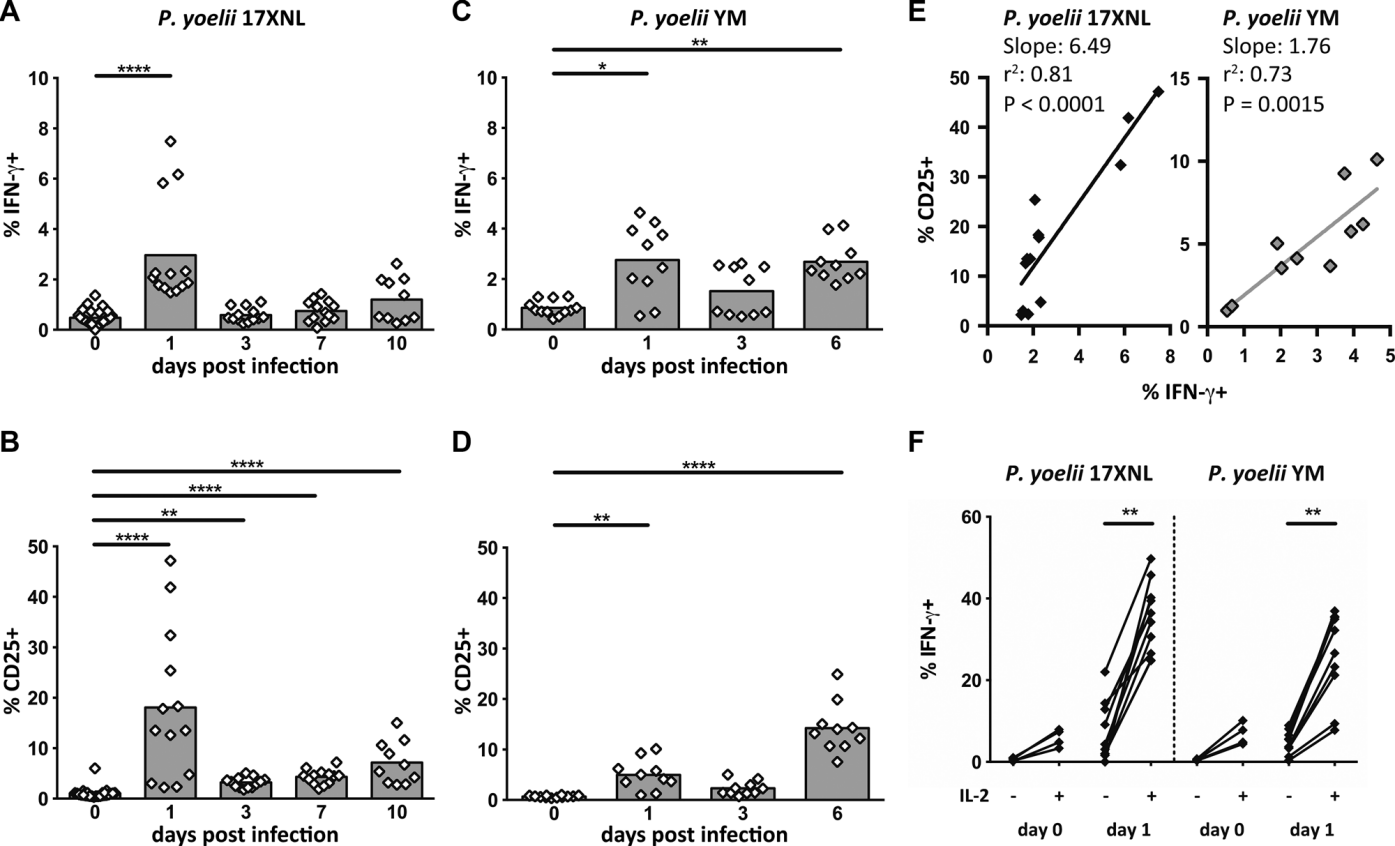
Association between NK‐cell expression of CD25 and IFN‐γ during lethal and nonlethal *P. yoelii* infection. (A–D) Mice were infected with (A, B) *P. yoelii* 17XNL or (C, D) *P. yoelii* YM and splenic NK cells were analyzed ex vivo in the first 6–10 days of infection for expression of IFN‐γ (A, C) and CD25 (B, D) by flow cytometry. The gating strategy was as shown in Figure 1C. (E) Correlation between ex vivo IFN‐γ and CD25 expression among NK cells collected on day 1 p.i. (*Py*17XNL filled diamonds; *Py*YM gray diamonds). (F) Percentage of splenic NK cells from uninfected (d0) or 1 day p.i. (d1) mice expressing IFN‐γ after coculture for 4 h with or without 100 ng/mL exogenous rIL‐2 (with Brefeldin A present for the last 3 h). Data points for individual mice, pooled from two (*Py*YM) or three (*Py*17XNL) independent experiments are shown (*n* = 10 for *Py*YM and *n* = 13 for *Py*17XNL). The Kruskal–Wallis plus Dunn's post‐test was used to compare experimental animals versus control (d0) animals (A–D) and the Wilcoxon test was used to compare IL‐2‐stimulated and unstimulated samples (F). *p < 0.05, **p < 0.01, ***p < 0.001, ****p < 0.0001.

To determine whether differences in the extent of NK‐cell activation simply reflected a difference in the timing of the NK response between the two infections, we compared NK‐cell expression of IFN‐γ, CD25, and CD122 in *Py*17XNL *and Py*YM infected mice over the first week of infection (Figs. 2 and 3). In mice with nonlethal *Py*17XNL infections, NK‐cell IFN‐γ production was maximal on day 1 p.i. (Figs. 2A and3A) and this coincided with the peak of CD25 expression (Figs. 2A and3B). Indeed, the proportion of NK cells expressing CD25 was very tightly correlated (*r* ^2^ = 0.81) with the proportion of NK cells producing IFN‐γ (Fig. 3E). By contrast, among mice with lethal *Py*YM infections, the NK‐cell IFN‐γ response was less robust on day 1 p.i. and was sustained until day 6 p.i. (when the mice were culled due to high parasitemia) (Fig. 3C). Strikingly, CD25 was upregulated much more gradually in *Py*YM‐infected mice, being maximal at day 6 p.i. (Fig. 3D). Nevertheless, there was a significant correlation between IFN‐γ production and CD25 expression in lethal *Py*YM infections (Fig. 3E).

To directly test the link between CD25 expression and IFN‐γ production, splenocytes from *P. yoelii* infected animals (24 h p.i.) and control (uninfected) animals were cultured in vitro with or without 100 ng/mL IL‐2 for 4 h and analyzed for IFN‐γ expression by flow cytometry (Fig. 3F). Splenic NK cells from uninfected animals expressed low levels of CD25 (Fig. 3B and D) and very few cells produced IFN‐γ irrespective of the presence or absence of IL‐2. By contrast, IFN‐γ production by NK cells from both *Py*17XNL‐infected and *Py*YM‐infected mice was markedly upregulated in the presence of IL‐2 with a trend, although not statistically significant, toward a higher IFN‐γ production by NK cells from *Py*17XNL‐infected mice than in NK cells from *Py*YM‐infected mice (Fig. 3F).

These data confirm recent reports of CD25 upregulation on murine NK cells during acute murine cytomegalovirus (MCMV) infection 23, indicate that CD25 expression might be causally associated with IFN‐γ production, and suggest (but do not prove) that a very rapid (24 h) and robust NK‐cell CD25/IFN‐γ response may be associated with successful resolution of *P. yoelii* infection.

### IL‐12 and IL‐18 synergize to induce CD25 expression on murine NK cells

**Figure 4:**
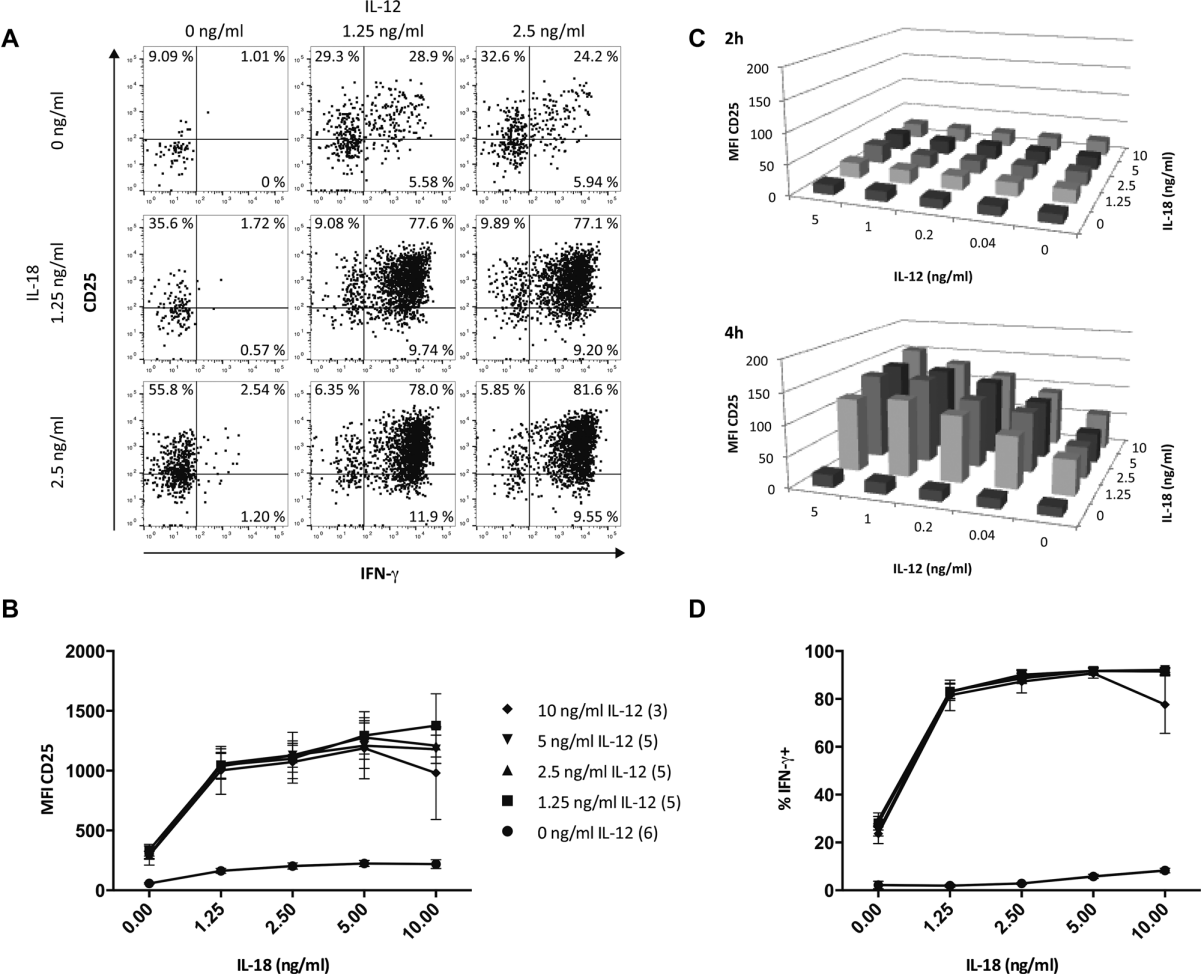
CD25 expression on NK cells is induced in vitro by IL‐12 and IL‐18. Splenocytes from naïve, uninfected mice were incubated with varying concentrations of recombinant IL‐12 and IL‐18 for 2–6 h and analyzed for CD25 and IFN‐γ expression by flow cytometry. (A) Representative dot plots of CD25 and IFN‐γ expression on NK cells after 6‐h stimulation with indicated concentrations of IL‐12 and IL‐18. The gating strategy was as shown in Figure 1C with additional gating on CD122 to ensure expression of the high‐affinity IL‐2R. Numbers in each quadrant denote the percentage of NK cells expressing CD25 and/or IFN‐γ. (B) Median MFI CD25 of NK cells after stimulation for 6 h with varying concentrations of IL‐12 and IL‐18. Data are from one of three independent experiments; the number of samples tested at each concentration is indicated in parentheses in the key. (C) CD25 expression levels (median MFI) on NK cells after 2‐ and 4‐h stimulation with varying concentrations of IL‐12 and IL‐18. Data from one of two independent experiments are shown (*n* = 5). (D) Proportion of IFN‐γ‐producing NK cells after stimulation for 6 h with varying concentrations of IL‐12 and IL‐18. Data are from one of three independent experiments. The number of samples tested at each concentration is indicated in the key of (B).

Since IL‐12 and IL‐18 are associated with induction of IFN‐γ in both mouse 24, 25, 26 and human 27, 28 NK cells, we wondered whether IL‐12 and/or IL‐18 might mediate this effect via induction of CD25. Splenic NK cells from uninfected mice were incubated for 2, 4, or 6 h with increasing concentrations of IL‐12, IL‐18, or IL‐12 plus IL‐18 and analyzed for IFN‐γ and CD25 expression by flow cytometry (Fig. 4A). In an initial experiment, as little as 1.25 ng/mL IL‐12 for 6 h was sufficient to induce CD25 expression on more than 50% of NK cells and further increasing the IL‐12 concentration did not significantly increase either the proportion of CD25^+^ cells (Fig. 4A) or the MFI of CD25 expression (Fig. 4B). Similarly, 1.25 ng/mL of IL‐18 was sufficient to induce CD25 on more than 30% of NK cells and 2.5 ng/mL IL‐18 induced CD25 expression on more than 50% of NK cells (Fig. 4A). Interestingly, IL‐12 alone induced a proportion of the CD25^+^ NK cells within the splenocyte population to express IFN‐γ, whereas IL‐18 alone did not induce IFN‐γ secretion (Fig. 4A). There was clear evidence of synergy between these two cytokines with 1.25 ng/mL of each inducing more than 80% of NK cells to express CD25 (Fig. 4A); the vast majority of these cells also expressed IFN‐γ.

To investigate further this synergy between IL‐12 and IL‐18, we examined upregulation of CD25 in response to even lower concentrations of IL‐12, over shorter incubation times (Fig. 4C). Although no CD25 upregulation was evident at 2 h, as little as 0.04 ng/mL IL‐12 in combination with 1.25 ng/mL IL‐18 was sufficient to markedly upregulate CD25 within 4 h (Fig. 4C, lower plot). In terms of IFN‐γ production, low concentrations of IL‐12 alone or IL‐18 alone had little effect on NK‐cell IFN‐γ production, but very low concentrations of IL‐12 and IL‐18 together (1.25 ng/mL of each) were able to induce more than 80% of NK cells to produce IFN‐γ (Fig. 4D).

### IL‐18 signaling is essential for CD25 expression and IFN‐γ production by NK cells during *P. yoelii* infection

**Figure 5:**
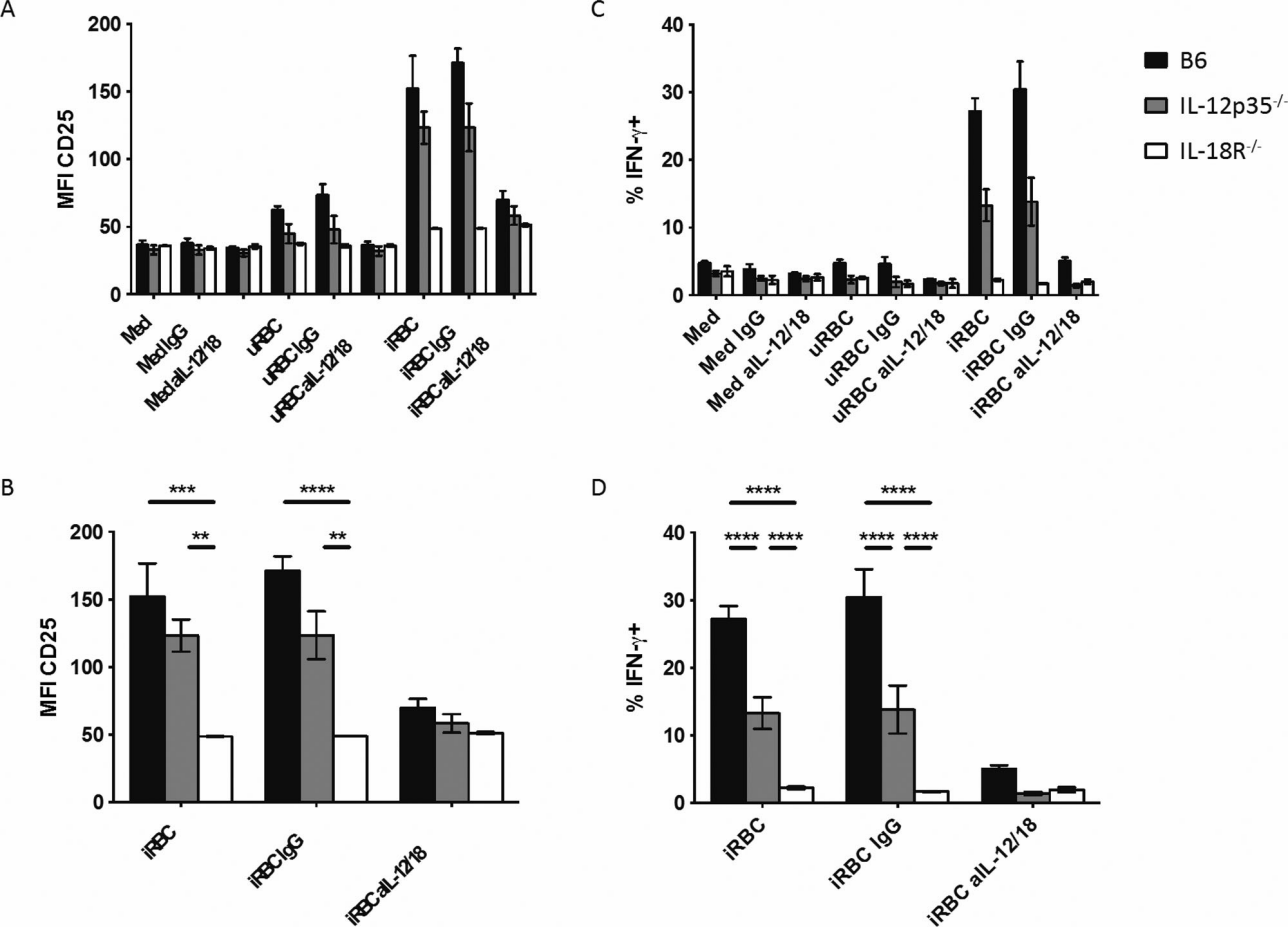
CD25 expression is induced by P. yoelii iRBC and is absolutely dependent upon IL‐18. (A–D) Splenocytes from WT C57BL/6 (B6; black bars), IL‐12p35^−/–^ (gray bars), and IL‐18R^−/−^ (white bars) mice were cultured for 19 h (with Brefeldin A present for the last 3 h) with uRBCs and iRBCs in the presence or absence of blocking antibodies to IL‐12 and IL‐18 or isotype control IgG antibody, and analyzed for (A, B) CD25 and (C, D) IFN‐γ expression. The entire dataset from one of two experiments is shown in (A) and (C) and statistical analyses for iRBC‐stimulated cells are shown in (B) and (D) (*n* = 3). ANOVA with Tukey's multiple comparison test was applied. *p < 0.05, **p < 0.01, ***p < 0.001, ****p < 0.0001.

As NK‐cell CD25 expression and IFN‐γ production are both induced during *P. yoelii* infection, IFN‐γ secretion is highly correlated with CD25 expression and enhanced by exogenous IL‐2, and CD25 is induced in vitro upon IL‐12 and IL‐18 stimulation, we hypothesized that *P. yoelii* iRBC would induce IL‐12 and/or IL‐18 secretion from monocytes or DCs and that these cytokines would then induce CD25 expression and IFN‐γ production from NK cells. To test this hypothesis, we cultured splenocytes from naïve (uninfected) C57BL/B6 mice, IL‐12 deficient mice (IL‐12p35^−/−^), or mice lacking the IL‐18 receptor (IL‐18R^−/−^) with *Py*17XNL iRBC or, as a control, uninfected RBCs (uRBC). In wild‐type (WT) C57BL/6 mice, uRBC induced modest upregulation of CD25, whereas iRBC induced a very marked (Fig. 5A) and highly significant (Fig. 5B) upregulation of CD25 on NK cells. CD25 upregulation by iRBC was modestly reduced in mice lacking IL‐12p35 indicating that NK‐cell activation is partly IL‐12 dependent. By contrast, iRBC‐induced upregulation of CD25 was completely ablated in IL‐18R^−/–^ mice, revealing an essential role for IL‐18 in upregulation of the high‐affinity IL‐2 receptor in response to *P. yoelii*. As confirmation of this, neutralizing antibodies to IL‐12 and IL‐18 completely ablated CD25 expression in WT and IL‐12p35 deficient mice (Fig. 5A and B).

To further investigate the functional relationship between expression of the high‐affinity IL‐2 receptor (CD25) and effector function (IFN‐γ), we attempted to neutralize IL‐2 in vitro in cocultures of iRBC and splenocytes using supposedly neutralizing monoclonal antibodies to murine IL‐2 (clones S4B6 and JES6; eBioscience, Hatfield, UK). Unfortunately, these experiments were unsuccessful, as blocking of IL‐2 in vitro gave anomalous and highly variable results (data not shown), possibly due to the tendency of both antibodies to potentiate IL‐2 signaling under certain conditions 29, 30, 31.

However, IFN‐γ  could be detected in over 30% of murine NK cells after 19‐h culture of naïve C57BL/6 mouse splenocytes with *P. yoelii* iRBC (Fig. 5C and D). By contrast, IFN‐γ production could not be detected at all in NK cells from IL‐18R^−/−^ mice and was significantly reduced in NK cells from IL‐12 deficient animals. In confirmation of this, IFN‐γ production was completely ablated by addition of blocking antibodies to IL‐12 and IL‐18 (Fig. 5C and D).

## Discussion

IFN‐γ is an important mediator of immune responses to blood‐stage malaria parasites 4, 5, 22, 32. NK cells are a significant source of IFN‐γ when human PBMC are cocultured in vitro with *P. falciparum* iRBC and may be the earliest source of this cytokine 7, 10. Although it is difficult to demonstrate directly a role for NK cells in protective immunity to malaria in humans, human NK cells have been shown to eliminate *P. falciparum* iRBCs in a humanized mouse model 11 and human population genetic studies are beginning to reveal associations between NK‐cell receptor genotype and outcome of malaria infection 33.

In experimental murine malaria infections, NK cells have been shown to contribute to protection against *P. yoelii* liver stages 18, blood‐stage *P. chabaudi* infection 13, 14, and nonlethal *P. yoelii* blood stages 5. IFN‐γ is detectable in splenic homogenates as early as 24 h after infection with nonlethal *P. yoelii* strains (but not after infection with lethal strains) 34; IFN‐γ  is also detectable (although at reduced concentrations) in athymic mice lacking T cells 34 but is reduced in animals depleted of NK cells by administration of anti‐asialo GM1 antibody 5, 34. Taken together, these data suggest a role for NK cells in the protective very early IFN‐γ response during murine malaria although—as anti‐asialo GM1 antibody also depletes some T‐cell populations 35 and IFN‐γ production was not investigated at the cellular level in these studies—the extent, timing, and significance of the NK‐cell contribution is unclear, as is the pathway of NK‐cell activation.

Here, we have shown substantial activation of murine splenic NK cells within 24 h of blood‐stage malaria infection with upregulation of CD25 (IL‐2Rα), the early activation marker CD69, and IFN‐γ. As expression of CD122 (IL‐2Rβ) is maintained on NK cells throughout the infection, this implies that a functional high‐affinity IL‐2 receptor is expressed on the activated, CD25^+^ cells. NK cells from mice with nonlethal *P. yoelii* infections responded more quickly and to a greater extent than did cells from mice with lethal infections, in agreement with previous findings that early NK‐cell responses correlate with favorable outcomes of murine malaria infections 5, 34. These data suggest, but do not prove, that early and robust NK‐cell responses contribute to initial control of blood‐stage malaria infections; NK‐cell depletion studies or NK cell specific gene deletion studies are needed to test this hypothesis.

In nonlethal *Py*17XNL infections, CD25 expression was very highly correlated with IFN‐γ production suggesting that these two responses might be causally associated. Indeed, 24 h after initiation of nonlethal malaria infection, splenic NK cells were hyper‐responsive to IL‐2, with exogenous IL‐2 inducing an approximate twofold increase in the proportion of NK cells producing IFN‐γ. Although much lower levels of CD25 were detected on splenic NK cells 24 h after infection with the lethal strain of *P. yoelii*, these also showed increased sensitivity to exogenous IL‐2 with substantially augmented IFN‐γ responses. These data suggest that the somewhat attenuated, and more variable, NK‐cell IFN‐γ responses observed in mice with lethal compared to nonlethal infections might result from limitations in the in vivo availability of IL‐2 as well as reliance on signaling via the low‐affinity (CD122) IL‐2R rather than the high‐affinity (CD25) receptor.

Although murine NK cells are known to respond to IL‐2 by proliferation and enhanced cytotoxic function 36, it has long been assumed that this response is mediated by the low‐affinity IL‐2R as studies in the early 1990s failed to detect expression of IL‐2Rα (CD25) in murine NK cells after in vivo administration of IL‐2 or IFN‐β or after infection with lymphocytic choriomeningitis virus 36. This view has, recently, been revised after the demonstration of transient expression of CD25 on NK cells from mice 2–3 days after infection with murine cytomegalovirus (MCMV); this was associated with approximately 100‐fold higher NK‐cell sensitivity to IL‐2 (measured by proliferation) 23. Optimal CD25 expression during MCMV infection required IL‐12/IL‐23p40 and signaling through the IL‐12R and STAT4; this may explain the lack of CD25 induction during lymphocytic choriomeningitis virus infection in which there is little if any IL‐12 response 37. Furthermore, CD25 could be induced on resting NK cells by a synergistic combination of 1 ng/mL each of IL‐12 and IL‐18 23. In confirmation of these observations, we have shown here that ∼1 ng/mL of IL‐12 in combination with ∼1 ng/mL IL‐18 induces maximal expression of CD25 on resting NK cells. However, our experiments build upon these published data to show that induction of CD25 is accompanied by IFN‐γ production, with more than 90% of the NK cells producing IFN‐γ under optimal conditions. These data are reminiscent of studies with human NK cells in which high concentrations of IL‐12 and IL‐18 can, in combination, induce high levels of IFN‐γ, over‐riding the need for other cytokines or contact‐mediated signals 27, 38. For human NK cells, IL‐2 synergizes with IL‐12 and IL‐18, markedly reducing the concentrations of IL‐12 and IL‐18 required to induce robust NK‐cell IFN‐γ responses (Wolf et al., unpubl. data). We have evidence for similar synergy among murine NK cells in that exogenous IL‐2 substantially enhanced IFN‐γ‐production by NK cells from *Py*17XNL‐ and *Py*YM‐infected mice and NK cells within splenocytes from *Rag2* KO mice (which lack T cell derived IL‐2) responded very poorly to *Py*17XNL‐ iRBCs (K. Stegmann, unpubl. data). Taken together, our data indicate that, during an infection, the timing of the NK‐cell response (and hence its contribution to controlling the infection) will be highly dependent upon the precise mix of cytokines being produced, their kinetics, and their respective concentrations. Further studies are now required to determine the optimal timing of these responses and their importance for controlling the initial stages of malaria parasite replication in the blood.

The robust NK‐cell response that we observed during *P. yoelii* infection also appears to be IL‐12 and IL‐18 dependent in that both CD25 and IFN‐γ were strongly upregulated when splenocytes from WT mice were cocultured with *P. yoelii* iRBC but this was blocked by neutralizing antibodies to IL‐12 and IL‐18. Interestingly, however, NK‐cell activation among splenocytes from IL‐12p35 deficient mice was only modestly reduced whereas NK cells from IL‐18R deficient mice were completely nonresponsive to iRBC. Preliminary experiments indicate that the NK cells themselves (rather than another cell type in the spleen) must express IL‐18R since NK cells from (CD45.2^+^) IL‐18R^−/−^ mice responded poorly to iRBC when cocultured with splenocytes from WT (CD45.1^+^) C57BL/6 mice (K. Stegmann, unpubl. data). Our data also suggest that the requirement for IL‐12 might be over‐ridden by provision of other cytokines (such as IL‐2 or IL‐15; notably addition of a very low concentration of IL‐15 to the culture medium was essential for NK survival in these experiments) but that the IL‐18‐mediated signals are essential and nonredundant. Previous in vivo studies have demonstrated an apparently essential role for IL‐12 in activation of NK cells to produce IFN‐γ during *P. chabaudi* 13, 14, 39 and *P. berghei* 40 infections but, although IL‐18 has been causally linked to IFN‐γ production and protection in *P. yoelii* and *P. berghei* infections 41, 42, this is the first demonstration of an essential role for IL‐18 in NK‐cell activation during murine malaria. Although the precise sources of IL‐12 and IL‐18 during very early *P. yoelii* infection remain to be determined, DCs and Ly6C^+^ CCR2^+^ inflammatory monocytes have been implicated as major sources of IL‐12 and IL‐18, respectively, during infections with other rodent malaria species 14, 40, 41.

In conclusion, we have shown that NK cells upregulate CD25 very early during experimental malaria infection, that induction of CD25 is entirely dependent upon IL‐18‐mediated signals, that extremely low concentrations of IL‐12 and IL‐18 can synergize to induce CD25, that CD25 expression is tightly correlated with induction of IFN‐γ, and that exogenous IL‐2 can enhance IFN‐γ production. These data suggest that, as for human NK cells responding to *P. falciparum*‐iRBCs 10, IL‐2‐mediated signaling pathways synergize with cytokine and/or contact‐mediated signals from myeloid cells to maximize the very early IFN‐γ response to blood‐stage malaria infection and that this response may be a crucial determinant of the eventual outcome of infection. Our data also imply that CD4^+^ or CD8^+^ T cells—the major source of IL‐2 in most systems—may also play a role in amplifying NK‐cell responses and thus that IL‐2 production by memory T cells may represent an important effector response during re‐infection or after vaccination.

## Materials and methods

### Animals and parasites

Female C57BL/6N (WT) mice were bred at the London School of Hygiene and Tropical Medicine or purchased from Charles River Laboratories (Margate, UK); IL‐12 deficient mice (IL‐12p35^−/−^) or mice lacking the IL‐18 receptor (IL‐18R^−/−^) were bred at the London School of Hygiene and Tropical Medicine and were shown to be free from prevalent bacterial, viral, fungal, and parasitic pathogens. All animal experiments were performed in accordance with the Animals (Scientific Procedures) Act of 1986 and were approved by the animal welfare and ethical review board of the London School of Hygiene and Tropical Medicine. Animals were housed in individually ventilated cages (three to five animals per cage) maintained in a 12 h light/12 h dark light cycle with environmental enrichment and received RM1 diet. Individual cages were randomly assigned to infection or control groups.

Cryopreserved *P. yoelii* parasites were thawed and passaged in vivo. Seven‐ to 10‐week‐old female mice were infected i.p. with 10^5^ iRBC; control mice were either naïve or treated with PBS or uRBC. Parasitemia was assessed every second day by the examination of Giemsa‐stained thin blood smears. Mice were killed by CO_2_ inhalation on selected days p.i. Single cell splenocyte suspensions were prepared by homogenization and sieving through a 70 μm cell strainer (BD Bioscience (BD), Oxford, UK). Samples were incubated in RBC lysis buffer (BD), washed, and resuspended in HBSS + 2% FCS (both Gibco Life Technologies, Paisley, UK).

### Flow cytometry

Cell preparations for phenotypic characterization directly ex vivo were processed and fixed within 2 h of collection, were kept on ice during processing, and were centrifuged at 4°C. Single cell suspensions were first stained with anti‐mouse CD122 (clone 5H4, eBioscience), anti‐mouse CD25 (clone PC64.5, eBioscience), anti‐mouse CD3 (clone 500A2, BD), anti‐mouse NKp46 (clone 29A1.4, BD), anti‐mouse CD69 (clone H1.2, eBioscience), anti‐mouse TRAIL (clone N2B2, eBioscience), anti‐mouse CD107a (clone 1D4B, eBioscience), and Live/Dead Fixable Near IR Dead Cell Stain Kit (Invitrogen) for 20 min at 4°C in the dark. After washing twice with HBSS + 2% FCS, cells were either fixed using 2% paraformaldehyde in PBS or permeabilized in BD Cytofix/Cytoperm for staining of intracellular cytokines for 20 min at 4°C. Before intracellular staining with anti‐mouse IFN‐γ (clone XMG1.2, eBioscience), anti‐mouse TNF (clone MP6‐XT22, eBioscience), and anti‐mouse Granzyme B (clone 16G6, eBioscience) for 20 min at 4°C in the dark, cells were washed twice with BD Perm/Wash buffer. After staining, cells were washed twice with BD Perm/Wash buffer and thereafter fixed using 2% paraformaldehyde in PBS for 20 min at 4°C. After a final wash, cells were resuspended in PBS. Samples were acquired using a BD LSRII flow cytometer and BD FACSDiva 6.0 (BD Bioscience). Data were analyzed using FlowJo for PC (TreeStar, Ashland, OR, USA).

### In vitro stimulation of splenocytes

In in vitro experiments, cell suspensions were stimulated with recombinant mouse IL‐2 (eBioscience) for 4 h and with IL‐12 (R&D, Abingdon, UK) and/or IL‐18 (MBL, Woburn, MA) for 2–6 h at 37°C at 5% CO_2_ with Brefeldin A (Sigma‐Aldrich, Dorset, UK) present for the last 3 h of incubation when IFN‐γ was analyzed alone or without Brefeldin A when CD25 and IFN‐γ were analyzed simultaneously. Stimulated cells were characterized by flow cytometry as described above.

### Coculture assay of splenocytes and RBC

In coculture experiments, isolated splenocytes of WT, IL‐12p35^−/−^, and IL‐18R^−/−^mice were cultured in RPMI1640 supplemented with 10% FCS, 100 U/mL penicillin/streptomycin, and 2 mM l‐glutamine (all Gibco Life Technologies) containing 2 ng/mL recombinant mouse IL‐15 (Peprotech, Rocky Hill, NJ, USA) with uRBC or *Py*17XNL iRBC at a ratio of 5 uRBC/iRBC per splenocyte. iRBCs were purified from blood obtained from *Py*17XNL‐infected WT animals by passage over a magnetic column (Miltenyi Biotech, Bergisch‐Gladbach, Germany). Blocking antibodies to IL‐12p35 (eBioscience) and IL‐18 (MBL) or control rat IgG1 or IgG2a (both eBioscience) were added at a concentration of 2.5 μg/mL. Cells were cultured at 37°C at 5% CO_2_ for 19 h with Brefeldin A present for the last 3 h of culture. Stimulated cells were characterized by flow cytometry as described above.

### Statistical analysis

Data are represented as means ± SEM. In Figure 3, data are presented as means of each day of infection showing individual data points and as individual data points for IL‐2 stimulation. Prism 6 (GraphPad Software, La Jolla, CA, USA) was used for preparation of figures and for statistical analysis. Kruskal–Wallis with Dunn's post‐test was used to compare experimental and control animals during the course of infection, Wilcoxon test was used to compare paired samples stimulated with IL‐2, and ANOVA with Tukey's multiple comparison test was used to compare coculture experiments using different transgenic mouse strains.

## Conflict of interest

The authors declare no financial or commercial conflict of interest.

**Abbreviations**
iRBC: infected red blood cell
MCMV: murine cytomegalovirus
p.i.: postinfection
uRBC: uninfected red blood cell

## Supporting information

As a service to our authors and readers, this journal provides supporting information supplied by the authors. Such materials are peer reviewed and may be re‐organized for online delivery, but are not copy‐edited or typeset. Technical support issues arising from supporting information (other than missing files) should be addressed to the authors.

Peer Review Correspondence
