# Supplementary material for: IL‐18‐induced expression of high‐affinity IL‐2R on murine NK cells is essential for NK‐cell IFN‐γ production during murine Plasmodium yoelii infection
Source: Eur J Immunol. 2015 Oct 21;45(12):3431–40. doi: 10.1002/eji.201546018 (PMC4982096; doi:10.1002/eji.201546018)
Supplement: Supplementary file 1 — Peer Review Correspondence [file EJI-45-3431-s001.pdf]

Peer review correspondence

European Journal of Immunology

IL-18-induced expression of high affinity IL-2R on murine NK cells is essential for NK-cell IFN-γ production during murine *Plasmodium yoelii* infection

Kerstin A. Stegmann, J. Brian De Souza and Eleanor M. Riley

Corresponding author: Eleanor M. Riley, Department of Immunology and Infection, Faculty of Infectious and Tropical Diseases, London School of Hygiene and Tropical Medicine, London, United Kingdom

---

Review Timeline:	Submission date:	4 August 2014
	First editorial decision:	18 September 2014
	First revision received:	17 December 2014
	Second editorial decision:	20 January 2015
	Second revision received:	31 January 2015
	Third editorial decision:	12 February 2015
	Third revision received:	19 August 2015
	Accepted:	23 September 2015

Handling Executive Committee members: Prof. Annette Oxenius

Please note that the correspondence below does not include the standard editorial instructions regarding preparation and submission of revised manuscripts, only the scientific revisions requested and addressed.

First Editorial Decision – 18 September 2014

Dear Ms. Stegmann, Dr. Riley,

Manuscript ID eji.201445096 entitled "Natural killer cell IFN-γ production during murine Plasmodium yoelii infection is entirely dependent upon IL-18-mediated induction of the high affinity IL-2 receptor" which you submitted to the European Journal of Immunology has been reviewed. The comments of the referees are included at the bottom of this letter.

A revised version of your manuscript that takes into account the comments of the referees will be reconsidered for publication. You will see that both referees 1 and 3 felt that some of your conclusions were not solidly supported by the data and as such require more proof. You should know that referee 1 felt

that this was grounds for rejection, and that our Executive Editor felt that it was important that you address the bulk of the concerns of these two referees with as much experimental data as possible.

You should also pay close attention to the editorial comments included below. *In particular, please edit your figure legends to follow Journal standards as outlined in the editorial comments. Failure to do this will result in delays in the re-review process.*

Please note that submitting a revision of your manuscript does not guarantee eventual acceptance, and that your revision will be re-reviewed by the referees before a decision is rendered.

If the revision of the paper is expected to take more than three months, please inform the editorial office. Revisions taking longer than six months may be assessed by new referees to ensure the relevance and timeliness of the data.

Once again, thank you for submitting your manuscript to European Journal of Immunology and we look forward to receiving your revision.

Yours sincerely,
Karen

On behalf of Prof. Annette Oxenius

Dr. Karen Chu
Editorial Office
European Journal of Immunology
e-mail: ejied@wiley.com
www.eji-journal.eu

****

Reviewer: 1
Comments to the Author
This paper by Riley and colleagues investigates the mechanisms regulating the production of IFNgamma by NK cells during blood stage murine malaria. IFNgamma is known to have an important protective role during this infection therefore defining which cells produce IFNgamma in vivo and potential underlying mechanisms is important. NK cells represent an important source of IFNgamma, also during malaria infection as previously suggested by this group and others. The current results provide a strong correlation between secretion of IFNgamma and expression of CD25. The authors demonstrate that IL-18 signaling is

European Journal of Immunology

required for CD25 expression by NK cells and -as already shown in other models- that IL-18 and IL-12 promote strong production of IFNgamma by NK cells. While some of these data are interesting, there are many flaws and the authors often overinterpreted their data:

1. The major message also in the title claiming that IFNgamma secretion by NK cells requires CD25 and IL-2 in vivo during P yoelii infection not formally demonstrated. IL-18 -and IL-12 to some extend- appears important for expression of these function and cell surface marker, and to promote NK cell activation (as known from multiple prior studies in different infections). The formal link between CD25/IL-2 and IFNgamma would require the use of CD25 KO NK cells or possibly IL-2 blocking experiments (though less clean).
2. Though it is clear that IL-18 is essential for NK cell activation, there are no experiments in this paper to discriminate whether IL-18 signals need to be NK cell intrinsic or not. These would have been simple experiments in vitro, for instance mixing WT and IL-18R KO splenocytes in comparable experiments as in Fig. 5.
3. Fig. 2 and 3: should be only one figure, Fig. 3 seems mostly building on analysis of data shown in Fig. 2. Peak production of IFNgamma occurs at 24 hours post infection, only very small frequencies of NK cell (max 6%) secrete the cytokine ex vivo.
The FACS dot plots showing expression of CD25 and IFNgamma ex vivo are not convincing. The difference of IFNgamma secretion by NK cells between lethal and non-lethal strain (even with data provided in panel E) to account for the respective outcomes is very speculative and may be removed. The panel F just suggest that NK cells have indeed been primed in vivo but no more.
4. Fig. 4: unclear how NK cells were purified? In general the figure legends and materials lack lots of details or are confusing.

Reviewer: 2
Comments to the Author
This manuscript is soundly based on previous knowledge of the roles and source of interferon (IFN)-gamma in lethal vs resolving murine malaria infection. It clearly demonstrates a novel aspect, namely the involvement of IL-2 in this process, as well as reinforcing the roles of IL-12 and IL-18 in the in vivo context.

There are a few issues that require clarification.

1. Figure 3. The statistical analysis of the data in panels A – D should be performed by ANOVA and a relevant post-test. If the data are non-parametric (likely) Kruskal-Wallis plus Dunn’s would be appropriate. The legend to the Figure seems to suggest that multiple comparisons were made using the Mann-Whitney test, which is not valid. This might explain why significant differences are indicated for comparisons when this seems unlikely from “eyeballing” the data, eg Panel A day 0 vs day 7; Panel B day 0 vs day 3.

European Journal of Immunology

2. The data in Fig 1D are presented as mean and range rather than mean ± SEM or SD – why? There are no statistical comparisons shown to justify the statements around lines 35 – 40 on page 5.

3. Is it sound to state (p6, lines 7 – 11) that "IFN-g production was not associated with upregulation of CD25 in lethal infections…" There was a clear and significant correlation, even though the slope was shallow.

4. p6, line 28: ".. with a trend towards a higher IFN-g production..." Was the difference significant or not?

5. p6, line 57: why not show these data?

6. Fig 2: the legend says that the data are "representative". How many experiments and how many mice showed similar outcomes?

Minor issues:
Quite a few errors of spacing or punctuation, commencing sentences with numerals, etc.

Reviewer: 3
Comments to the Author
The studies reported in this manuscript investigated the pathways involved in NK cell activation for IFN-γ secretion in mice infected with non-lethal 17XNL compared to lethal YM P. yoelii blood stage parasites. The major findings are: 1) CD25 expression is up-regulated on NK cells early during infection; 2) CD25 induction on NK cells requires IL-18-dependent signaling; 3) low concentrations of IL-18 and IL-12 synergise to induce CD25 up-regulation on NK cells; and 4) CD25 expression significantly correlates with NK cell secretion of IFN-γ and resolution of infection in mice with non-lethal 17XNL infection. Based on the data presented, the authors suggest that IL-2 mediated signaling synergize with cytokine and/or contact mediated signals from myeloid cells to induce NK cell IFN-γ secretion early during blood stage malaria infection and that the likely source of IL-2 may be CD4+ or CD8+ T cells.

The experiments are well designed and were executed in a technically sound manner, the data presented are highly reproducible, and data from ex vivo as well as in vivo experiments are provided. However, the data are primarily descriptive and largely reproduce the in vitro findings in NK cell IFN-γ responses to P. falciparum-infected RBC in PBMC described earlier by the authors. Additional experiments are required to: 1) identify the myeloid cell(s) or other cell types that secrete the cytokine signals that is IL-18 and IL-12; 2) determine if NK cell activation in P. yoelii-infected mice requires contact-dependent interactions with myeloid or other cell types in addition to cytokines; and 3) to identify the cell or cell type that provides the

early source of IL-2 for NK cell activation in P. yoelii infected mice. The addition of the data from such experiments would support the authors' contentions regarding the role of IL-2 in IFN-γ secretion by NK cells during P. yoelii infection as well as the major source of IL-2 involved in inducing IFN-γ secretion by activated NK cells in this system.

Specific comments and concerns

1. Fig. 1C and page 5, lines 28-41: the text and order of the panels should be arranged in a similar order to make it easier for readers to follow.
2. Fig. 1D: the format of the table is unusual and not easy to interpret. The columns should be ordered in a similar manner and the important data highlighted.
3. Figs. 2 and 3 and page 5 line 44 to page 6 line 12, what is the rationale for determining CD122 expression on NK cells? Wouldn't it be more relevant to look at a marker of NK cell activation?
4. The authors compare NK cell activation in their malaria model to findings in mice with LCMV and MCMV infections in the Discussion. On page 6 lines 33-39, the authors indicate that their data confirms reports of CD25 expression of activated NK cells during "acute" infection. The acute infection should be identified since the reference cited refers to acute virus infection.

<u>First revision – authors' response – 17 December 2014</u>

Reviewer: 1

1. The major message also in the title claiming that IFNgamma secretion by NK cells requires CD25 and IL-2 in vivo during P yoelii infection not formally demonstrated. IL-18 -and IL-12 to some extend- appears important for expression of these function and cell surface marker, and to promote NK cell activation (as known from multiple prior studies in different infections). The formal link between CD25/IL-2 and IFNgamma would require the use of CD25 KO NK cells or possibly IL-2 blocking experiments (though less clean).

This is a very important point and we agree that formal demonstration that the NK cell responses to malaria are IL-2-dependent is required. We therefore conducted additional experiments to determine whether blocking antibodies to IL-2 ablate the NK cell response. We observed an approximately 30% reduction in NK cell activation in the presence of anti-IL-2 antibodies but the experiment has only been performed once and it is not feasible to repeat the assays as Dr Stegmann has left the lab and my UK animal license for this work has expired. I do not feel it is appropriate to include these preliminary data in the manuscript but I have commented on the results of our preliminary experiment in the discussion and made it clear that additional experiments would be needed to confirm the hypothesis (pages 10, 11).

European Journal of Immunology

In fact, these additional data, combined with the reanalysis of the data in Figure 3 (see Reviewer 2, point 1), reinforce the conclusion expressed in the Discussion of the paper (but perhaps not adequately reflected in the Title or Abstract) that IL-2 signalling enhances the NK cell response but is not essential - the response is not completely ablated in the presence of anti-IL-2; in PyYM-infected mice there is a low level of IFN-γ secretion despite a complete lack of CD25 upregulation on day 1 pi (Figure 3D); and we see enhancement of the response to iRBC in the presence of exogenous IL-2 (Fig 3F). I have amended the Title of the manuscript, the Abstract and the Discussion (page 11) to better reflect this interpretation of the data.

2. Though it is clear that IL-18 is essential for NK cell activation, there are no experiments in this paper to discriminate whether IL-18 signals need to be NK cell intrinsic or not. These would have been simple experiments in vitro, for instance mixing WT and IL-18R KO splenocytes in comparable experiments as in Fig. 5.

We agree with this comment and have conducted preliminary experiments mixing purified IL-18R KO NK cells with wild type splenocytes. Again, I do not feel that the data we have are complete enough to add to the manuscript but I have commented on the results of our preliminary experiment in the discussion of the manuscript (bottom of page 11).

3. Fig. 2 and 3: should be only one figure, Fig. 3 seems mostly building on analysis of data shown in Fig. 2.

The reviewer is correct that the data in Figures 2 and 3 are linked (Figure 2 shows the example FACS plots on which the data in Figure 3 are based) but we prefer to keep them as separate Figures as combining them into a single figure would reduce the size of the FACS plots to an extent that they would be impossible to see on the printed page. However, we are willing to reconsider this if the Editor wishes us to. One option might be to move Figure 2 to supplementary online material?

The FACS dot plots showing expression of CD25 and IFNgamma ex vivo are not convincing. The difference of IFNgamma secretion by NK cells between lethal and non-lethal strain (even with data provided in panel E) to account for the respective outcomes is very speculative and may be removed. The panel F just suggest that NK cells have indeed been primed in vivo but no more.

We agree, on reflection, that the differences in IFN-γ responses between lethal and non lethal malaria infections are not perhaps as convincing as we originally thought: although the response is much more consistent in the PyNL-infected mice and some animals have very high levels of IFN-γ production the median values are similar. On the other hand, the reanalysis of the CD25 data (see Reviewer 2, point 1)

European Journal of Immunology

confirms highly significant induction of CD25 in PyNL mice on day 1pi but no significant induction of CD25 in PyYM mice until day 6pi. We have edited the text (bottom of page 5, top of page 6) to reflect this.

4. Fig. 4: unclear how NK cells were purified? In general the figure legends and materials lack lots of details or are confusing.

NK cells were not purified. Data in Figure 4 was obtained from in vitro culture of mixed splenocytes. We apologise for the lack of clarity of the figure legends. We have reviewed and revised all the figure legends to ensure that all necessary information is included.

Reviewer: 2

1. Figure 3. The statistical analysis of the data in panels A – D should be performed by ANOVA and a relevant post-test. If the data are non-parametric (likely) Kruskal-Wallis plus Dunn's would be appropriate. The legend to the Figure seems to suggest that multiple comparisons were made using the Mann-Whitney test, which is not valid. This might explain why significant differences are indicated for comparisons when this seems unlikely from "eyeballing" the data, eg Panel A day 0 vs day 7; Panel B day 0 vs day 3.

We thank the reviewer for this very helpful comment. The data have been reanalysed as suggested and the statistical analysis section of the materials and methods has been updated (Page 15).

The re-analysis revealed that upregulation of IFN-γ in the non-lethal PyNL infection at 24h post infection was more robust than previously realised. On the other hand, in the lethal PyYM infection, CD25 upregulation at 24h post infection was no longer statistically significant, confirming our initial impression that CD25 expression is delayed in PyYM infections compared to PyNL infections.

2. The data in Fig 1D are presented as mean and range rather than mean ± SEM or SD – why?

We have added the SEM values to Figure 1D.

There are no statistical comparisons shown to justify the statements around lines 35 – 40 on page 5.

We assume this statement refers to data shown in Figure 3? In which case, the statistical information is shown in the Figure and it seems unnecessary to repeat it in the text?

European Journal of Immunology

3. Is it sound to state (p6, lines 7 – 11) that “IFN-g production was not associated with upregulation of CD25 in lethal infections…” There was a clear and significant correlation, even though the slope was shallow.

We completely agree. Although the correlation is significant the slope of the line as shown in the original figure suggested that there was very little increase in IFN-γ production irrespective of CD25 expression. However, when the data for the two infections are plotted on separate axes, the association is much more obvious. We have revised Figure 3E and edited the text (top of page 6) to reflect this.

4. p6, line 28: “.. with a trend towards a higher IFN-g production...” Was the difference significant or not?

The difference was not statistically significant. This is now clearly stated on page 6.

5. p6, line 57: why not show these data?

Our original wording of this sentence was confusing, for which we apologise, although it is true that the 2h data was not shown in the original Figure. We have added the 2h data to Fig 4B. Data for 4h is shown in Fig 4B and for 6h in Figure 4C and 4D. The Figure has been changed and the text has been clarified (bottom of Page 6).

6. Fig 2: the legend says that the data are “representative”. How many experiments and how many mice showed similar outcomes?

Figure 2 shows representative plots of lethal and non-lethal malaria infection, which are summarized in Figure 3, we apologise for not making this clear in the legend, the necessary information has been added. All figure legends have been revised to include this information.

Minor issues:
Quite a few errors of spacing or punctuation, commencing sentences with numerals, etc.

We apologise for these errors. The manuscript has been carefully checked and revised.

Reviewer: 3

Additional experiments are required to: 1) identify the myeloid cell(s) or other cell types that secrete the cytokine signals that is IL-18 and IL-12; 2) determine if NK cell activation in P. yoelii-infected mice requires contact-dependent interactions with myeloid or other cell types in addition to cytokines; and 3) to identify

European Journal of Immunology

the cell or cell type that provides the early source of IL-2 for NK cell activation in P. yoelii infected mice. The addition of the data from such experiments would support the authors' contentions regarding the role of IL-2 in IFN-γ secretion by NK cells during P. yoelii infection as well as the major source of IL-2 involved in inducing IFN-γ secretion by activated NK cells in this system.

We agree that further studies are required to verify the sources of accessory cytokines and the role of contact mediated signals but we respectfully suggest that this would require extensive additional experimentation that falls outside the scope of this particular paper. Nevertheless, sources of IL-12 and IL-18 have been reported for other murine malaria infections and this is now discussed and referenced (Page 12; additional references 40 and 41).

Specific comments and concerns
1. Fig. 1C and page 5, lines 28-41: the text and order of the panels should be arranged in a similar order to make it easier for readers to follow.

We agree and have re-ordered the plots in Figure 1C to reflect the order in which the parameters are discussed in the text. Thank you for this suggestion.

2. Fig. 1D: the format of the table is unusual and not easy to interpret. The columns should be ordered in a similar manner and the important data highlighted.

We agree that the lay out of the Table was unusual but we wanted to have the two colour coded columns next to each other so that they could be easily compared. However, we have revised the Table in accordance with the reviewer's suggestion. The important data is indeed highlighted by the use of different colours (as a heat map) as is now widely used for comparing large numbers of parameters – green for increased expression and yellow for no change (we would have used red for down regulation, but did not see any examples of this).

3. Figs. 2 and 3 and page 5 line 44 to page 6 line 12, what is the rationale for determining CD122 expression on NK cells? Wouldn't it be more relevant to look at a marker of NK cell activation?

CD122 (IL-2Rβ) is constitutively expressed on NK cells and, together with the common γ chain, forms the low affinity receptor for IL-2. Expression of CD25 (IL-2Rα) converts the intermediate receptor into the high affinity receptor. Importantly, CD122 - but not CD25 – mediates IL-2 signalling. Thus, expression of fully functional high affinity IL-2Rs requires continued expression of CD122 as well as upregulation of CD25. We therefore included CD122 in our staining panels to ensure that cells expressing CD25 were in fact expressing fully functional high affinity IL-2R. Our data show sustained expression of CD122 throughout

the infection, confirming that CD25 upregulation is functionally relevant. This is now made clear on page 9. We used CD69 as an activation marker and this was strongly upregulated (as shown in Figure 1).

4. The authors compare NK cell activation in their malaria model to findings in mice with LCMV and MCMV infections in the Discussion. On page 6 lines 33-39, the authors indicate that their data confirms reports of CD25 expression of activated NK cells during "acute" infection. The acute infection should be identified since the reference cited refers to acute virus infection.

We have clarified that the acute infection referred to is murine cytomegalovirus (Page 6).

Second Editorial Decision – 20 January 2015

Dear Prof. Riley,

Thank you for your patience while we found the time to evaluate the re-review of your revised manuscript ID eji.201445096.R1 entitled "IL-18 induces expression of the high affinity IL-2 receptor on murine NK cells and is essential for NK cell IFN-γ production during murine Plasmodium yoelii infection" which you had submitted to the European Journal of Immunology. Your manuscript has been re-reviewed and the comments of the referees are included at the bottom of this letter.

Unfortunately, referee 1 was not satisfied with the revisions made and feels that single experiments, the data of which you cannot show for obvious reasons, is not acceptable. This referee recommended that we reject the paper; however the Executive Editor agrees this is a tricky situation and while we cannot expect you to perform all of the experiments requested by this referee, again for obvious reasons, she does feel that an effort should be made to make the novel point of your story as solid as possible - see her comments below. The journal does not encourage multiple rounds of revision and we strongly encourage you to revise your paper as outlined by the Executive Editor in this final round of revision.

You should also pay close attention to the editorial comments included below. *In particular, please edit your figure legends to follow Journal standards as outlined in the editorial comments. Failure to do this will result in delays in the re-review process.*

Please note that submitting a revision of your manuscript does not guarantee eventual acceptance, and that your revision will be re-reviewed by the referees before a decision is rendered.

If the revision of the paper is expected to take more than three months, please inform the editorial office. Revisions taking longer than six months may be assessed by new referees to ensure the relevance and timeliness of the data.

Once again, thank you for submitting your manuscript to European Journal of Immunology and we look forward to receiving your revision.

Yours sincerely,
Karen Chu

On behalf of Prof. Annette Oxenius

Dr. Karen Chu
Editorial Office
European Journal of Immunology
e-mail: ejied@wiley.com
www.eji-journal.eu

****

Reviewer: 1
Comments to the Author
Some additional experiments have been done to address the most essential comments of the reviewers but all are "not shown" , one-time experiments that are not even provided as personal communication to the reviewers. Thus I feel the revised manuscript by Stegmann et al. suffers the same limitations as in the initial version. I also feel lots of data are over-interpreted and I am not convinced about the novelty of the findings as it stands, specifically:

1. The major link with IL2 is still unclear, even the authors admit that only a 30% loss in NK cell activation can be measured. It is also unclear how activation was measured, was this IFNgamma? No data are provided, even for the reviewer's assessment. Why is CD25 only shown in histogram and not dot plots (Fig 1&2)? Histograms are not convincing, similarly to the claimed differences in TNF, CD107 etc). The Y axis in fig 1 cannot be NKp46, there is only one dimension here.

2. The fact that differences in IFNgamma production by NK cells may explain why mice infected by non-lethal P yoelii XNL survive compared to mice infected with lethal Py YM XL is far from being convincing (see figure 3). Differences are truly really marginal, and mostly seen at a one late time point. Here, the

only solid conclusion could be that NK cells are activated and secrete some IFNgamma (less than 5% of NK though).

3. The fact that NK cells can be activated by IL12/18 is not novel and has been shown in many infections previously. The only potentially new finding hypothesized by the authors is related to IL-18 inducing CD25 expression on NK cells, still is not really shown. These experiments are simple in vitro experiments and could be done very easily even though the lead author have left the lab.

Reviewer: 3
Comments to the Author
I am fully satisfied with the revised version of the manuscript.

Executive Editor
Although I can understand that the leaving of the first author of the paper makes it a bit difficult to do all the requested experiments suggested by reviewer 1, I also think that this is not a good reason why one should accept a manuscript without asking for the solid additional data. I would suggest that the conclusions of the manuscript have to be adapted such that they are really backed-up with solid data (not single experiments) and that at least point 3 of reviewer 2 ("The fact that NK cells can be activated by IL12/18 is not novel and has been shown in many infections previously. The only potentially new finding hypothesized by the authors is related to IL-18 inducing CD25 expression on NK cells, still is not really shown. These experiments are simple in vitro experiments and could be done very easily even though the lead author have left the lab") needs to be addressed experimentally.

Second revision – authors' response – 31 January 2015

Reviewer: 1

1. The major link with IL2 is still unclear, even the authors admit that only a 30% loss in NK cell activation can be measured. It is also unclear how activation was measured, was this IFNgamma? No data are provided, even for the reviewer's assessment. Why is CD25 only shown in histogram and not dot plots (Fig 1&2)? Histograms are not convincing, similarly to the claimed differences in TNF, CD107 etc). The Y axis in fig 1 cannot be NKp46, there is only one dimension here.

European Journal of Immunology

We apologise for the lack of clarity in our original response. The 30% reduction in NK activation after IL-2 blockade was indeed measured by IFN-γ production, and although this was clearly stated in the revised manuscript (on page 11) we did not make it crystal clear in the response letter.
This reviewer objects to showing example flow cytometry plots as histograms. We find this surprising as we believe our data presentation to be standard format and best practice for reporting flow cytometry data. That is, that we use a histogram when a single parameter is being shown so that the absolute number of events (count) can be shown as well as the fluorescence intensity, and that we use dot plots when we wish to show two parameters simultaneously. However, if the Editor would like us to change the presentation of the data, we will of course oblige.
We apologise profusely for the mislabelling of the axis in several of the histograms in Figure 1. This was a classic “cut and paste” error and we are most grateful to the reviewer for spotting it. The axes have been corrected.

2. The fact that differences in IFNgamma production by NK cells may explain why mice infected by non-lethal P yoelii XNL survive compared to mice infected with lethal Py YM XL is far from being convincing (see figure 3). Differences are truly really marginal, and mostly seen at a one late time point. Here, the only solid conclusion could be that NK cells are activated and secrete some IFNgamma (less than 5% of NK though).

The reviewer made this same comment in his/her previous review: “The difference of IFNgamma secretion by NK cells between lethal and non-lethal strain (even with data provided in panel E) to account for the respective outcomes is very speculative and may be removed”.

We agreed with this comment and edited the manuscript accordingly in our first revision: “We agree, on reflection, that the differences in IFN-γ responses between lethal and non lethal malaria infections are not perhaps as convincing as we originally thought: although the response is much more consistent in the PyNL-infected mice and some animals have very high levels of IFN-γ production the median values are similar. On the other hand, the reanalysis of the CD25 data (see Reviewer 2, point 1) confirms highly significant induction of CD25 in PyNL mice on day 1pi but no significant induction of CD25 in PyYM mice until day 6pi. We have edited the text (bottom of page 5, top of page 6) to reflect this.”

We are not sure what more we can do to satisfy the reviewer as we feel we have effectively dealt with the comment? We would note however, that the 5% of NK cells actively secreting IFN-γ is immediately ex vivo without further in vitro stimulation, which represents a biologically significant – and not, as the reviewer implies, a negligible - in vivo response.

3. The fact that NK cells can be activated by IL12/18 is not novel and has been shown in many infections previously. The only potentially new finding hypothesized by the authors is related to IL-18 inducing CD25

European Journal of Immunology

expression on NK cells, still is not really shown. These experiments are simple in vitro experiments and could be done very easily even though the lead author have left the lab.

Again, we are at a loss to understand this comment. In Figure 4 we show a detailed time course and dose response curves for upregulation of CD25 on NK cells by IL-12, IL-18 and a combination of both cytokines together. These data are shown as dot plots of the % of CD25+ cells in Figure 4A, and as MFI of CD25 expression in Figs 4B and 4C. This is entirely novel data for murine NK cells.

Moreover, we show in Figure 5 that activation of NK cells by malaria infected red blood cells (in terms of both CD25 expression and IFN-γ secretion) is entirely dependent upon expression of a functional IL-18 receptor. This is entirely novel data.

Reviewer: 3
Comments to the Author
I am fully satisfied with the revised version of the manuscript.
Thank you!

Executive Editor
Although I can understand that the leaving of the first author of the paper makes it a bit difficult to do all the requested experiments suggested by reviewer 1, I also think that this is not a good reason why one should accept a manuscript without asking for the solid additional data. I would suggest that the conclusions of the manuscript have to be adapted such that they are really backed-up with solid data (not single experiments) and that at least point 3 of reviewer 2 ("The fact that NK cells can be activated by IL12/18 is not novel and has been shown in many infections previously. The only potentially new finding hypothesized by the authors is related to IL-18 inducing CD25 expression on NK cells, still is not really shown. These experiments are simple in vitro experiments and could be done very easily even though the lead author have left the lab") needs to be addressed experimentally.

We thank you for your understanding and we entirely agree that all the statements we make must be supported by solid data. We believe that the conclusions of the manuscript are backed up by solid data. Where additional data would be required to support a conclusion we explicitly acknowledge this and we have taken great care not to draw conclusions that we cannot support.

Specifically, we conclude that:
1. NK cells are activated within 24h of Py17XNL blood-stage infection and this response is blunted and delayed during PyYM infection: Data in Figures 1 and 3

2. CD25 expression and IFN-γ production are highly correlated: Figure 3E.
3. IL-18 signalling is essential for induction of CD25: Figure 5
4. IL-18 synergises with IL-12 to enhance CD25 expression: Figure 4
5. Py17XNL-infected erythrocytes induce NK cell CD25 expression and IFN-γ production in a manner that is completely IL-18 and partially IL-12 dependent: Figure 5
6. IFN-γ production is enhanced by IL-2: Figure 3F

We hope that our comments above have addressed your concerns, particularly with respect to the data on IL-18 and CD25 expression, which we believe is robustly supported by the data we have presented in the original manuscript and in the revised manuscript (Figures 4 and 5).

Third Editorial Decision – 12 February 2015

Dear Prof. Riley,

Thank you for patience while the Executive Editor and I had a closer look at your revised manuscript ID eji.201445096.R2 entitled "IL-18 induces expression of the high affinity IL-2 receptor on murine NK cells and is essential for NK cell IFN-γ production during murine Plasmodium yoelii infection" which you had submitted to the European Journal of Immunology. We wanted to evaluate your responses to the referees in-house and this took a bit of time.

We have had a look through your revision and feel that we overlooked that you had addressed some of referee 1's points already, and apologise that we insisted on more data. However, we feel that the novel part of the paper could be made a lot more convincing with better data presentation; the Editor and I have gone through and our suggestions for these minor revisions can be found in the comments below.

You should also pay close attention to the editorial comments included below. *In particular, please edit your figure legends to follow Journal standards as outlined in the editorial comments. Failure to do this will result in delays in the re-review process.*

Once again, thank you for submitting your manuscript to European Journal of Immunology. We look forward to receiving your revision.

Yours sincerely,
Karen Chu

on behalf of Prof. Annette Oxenius

Peer review correspondence

Dr. Karen Chu
Editorial Office
European Journal of Immunology
e-mail: ejied@wiley.com
www.eji-journal.eu

****

Executive Editor comments:

1. I think the authors have clearly shown that IL-18 induces CD25 expression in NK cells; so comment No. 3 by reviewer 1 was not fair and we should accept Figure 4.

2. I agree with reviewer 1 that the novelty of the data is not that IL-18 and IL-12 can induce IFNg production in NK cells (this has been shown many times before) but that this would operate via CD25 upregulation and IL-2 signaling in NK cells. I think it is really a pity that this conclusion of the authors is not supported by experimental data (they only mention on page 11 of the discussion the preliminary data with IL-2 neutralization in vitro which reduced IFN-g production by 30% - which would not really support a very strong importance of the IL-2/CD25 axis). I think these data need to be included and once more repeated (which does not seem very difficult to me, even for a new student in the lab). This is the least "new data" the authors should provide - we are not asking of doing in vivo infection experiments with CD25-deficient NK cells (which would be the optimal read-out).

3. The authors' statement "Py17XNL-infected erythrocytes induced NK cell CD25 expression and IFN-γ production in a manner that is completely IL-18 and partially IL-12 dependent" in the abstract is perhaps a bit misleading as they show with recombinant cytokines in vitro that CD25 can also be upregulated by IL-12 alone.

4. Figure 2: Here the authors could and should gate on the CD25+ and the CD25- cells and assess whether IFN-g production is enriched (or even exclusively found) in the CD25+ population.

5. Figure 3: The authors should plot % CD25 in B and D and correlate in E % CD25 with % IFN-g (or alternatively MFI CD25 and MFI IFNg).

6. Figure 4: Again it would be nice to see the FACS plots of IFN-g production and whether or not IFN-g-producing cells are enriched in the CD25+ cells.

European Journal of Immunology

7. It is unclear, based on the weak ex vivo IFN staining in NK cells, why theauthors have not blocked in vivo or a least during the harvesting of the cells cytokine secretion by BFA - or have they done it and not mentioned it?

Third revision – authors' response – 19 August 2015

Executive Editor comments:

1. I think the authors have clearly shown that IL-18 induces CD25 expression in NK cells; so comment No. 3 by reviewer 1 was not fair and we should accept Figure 4.

Thank you.

2. I agree with reviewer 1 that the novelty of the data is not that IL-18 and IL-12 can induce IFNg production in NK cells (this has been shown many times before) but that this would operate via CD25 upregulation and IL-2 signaling in NK cells. I think it is really a pity that this conclusion of the authors is not supported by experimental data (they only mention on page 11 of the discussion the preliminary data with IL-2 neutralization in vitro which reduced IFN-g production by 30% - which would not really support a very strong importance of the IL-2/CD25 axis). I think these data need to be included and once more repeated (which does not seem very difficult to me, even for a new student in the lab). This is the least "new data" the authors should provide - we are not asking of doing in vivo infection experiments with CD25-deficient NK cells (which would be the optimal read-out).

We have repeated the IL-2 neutralisation assays two more times (making three experiments in total), using two different neutralising monoclonal antibodies (anti-IL-2 JES6 and anti-IL-2S46B). Both antibodies are sold, by a number of companies, as suitable for in vitro neutralisation. However, in all experiments and with both antibodies we obtained highly anomalous results that varied from experiment to experiment and with differing concentrations of antibody. The one consistent effect that we saw was that anti-IL-2 antibodies acted additively with anti-IL-12 and anti-IL-18 antibodies to supress NK cell IFN-γ production. Whilst these data are highly consistent with our hypothesis, we not comfortable with using IL-2 blockade to confirm our data on the role of IL-2 in NK cell activation.

On further investigation, it appears that both of these anti-IL-2 antibodies have been found, under certain conditions, to potentiate rather than block IL-2 signalling, most likely by increasing the half-life of bioavailable IL-2. For example:

For JES6: Lee SY,et al. Interleukin-2/anti-interleukin-2 monoclonal antibody immune complex suppresses collagen-induced arthritis in mice by fortifying interleukin-2/STAT5 signalling pathways. Immunology. 2012 Dec;137(4):305-16.

For S46B: Phelan JD, Orekov T, Finkelman FD. Cutting edge: mechanism of enhancement of in vivo cytokine effects by anti-cytokine monoclonal antibodies. J Immunol. 2008 Jan 1;180(1):44-8.

For both JES6 and S4B6: Gasteiger G, Hemmers S, Bos PD, Sun JC, Rudensky AY. IL-2-dependent adaptive control of NK cell homeostasis. J Exp Med. 2013 Jun 3;210(6):1179-87.

For reasons detailed in our previous response, we are not in a position to carry out in vivo experiments with CD25-deficient NK cells, as suggested. We were, however, able to perform one in vitro experiment with spleen cells from RAG-2 deficient mice (which lack T cell derived IL-2) and found that their NK cells responded very poorly to malaria infected RBC in vitro compared to wild type B6 mice, but we cannot rule out that this is due to other effects of T cell and B cell deficiency. We have amended the text of our manuscript on page 8 to make this clear.

We are very disappointed not to be able to close the final loop in our argument in the way that you would like, but we would draw your attention to the considerable volume of data that we have presented to support our arguments. Specifically, we have shown that

1. CD25 expression on NK cells is markedly upregulated during malaria infection (Figure 1)
2. CD25 expression and IFN-γ production are highly correlated (Figure 3E).
3. IFN-γ production is enhanced by exogenous IL-2 (Figure 3F)
4. Malaria-infected erythrocytes induce NK cell CD25 expression and IFN-γ production in a manner that is completely IL-18 and partially IL-12 dependent: Figure 5

Moreover, we have been extremely careful in the way we have reported our data not to make any claims for which we do not have the empirical data.

3. The authors' statement "Py17XNL-infected erythrocytes induced NK cell CD25 expression and IFN-γ production in a manner that is completely IL-18 and partially IL-12 dependent" in the abstract is perhaps a bit misleading as they show with recombinant cytokines in vitro that CD25 can also be upregulated by IL-12 alone.

We beg to disagree. It is true that very high (completely non-physiological) concentrations of exogenous IL-12 can induce CD25 expression, but this is not what we are saying. The crucial piece of data supporting the statement that "Py17XNL-infected erythrocytes induced NK cell CD25 expression and IFN-γ

production in a manner that is completely IL-18 …dependent" is shown in Figure 5: specifically, NK cells lacking the IL-18R are completely nonresponsive to malaria infected red blood cells. We stand by our statement and believe it is fully supported by the data.

4. Figure 2: Here the authors could and should gate on the CD25+ and the CD25- cells and assess whether IFN-γ production is enriched (or even exclusively found) in the CD25+ population.

Unfortunately, in these in vivo experiments, CD25 and IFN-γ were included in two separate flow cytometry staining panels so it is not possible to directly analyse IFN-γ production by CD25 status. We cannot repeat these experiments as our license for animal work has expired. However, we have done this analysis for the in vitro studies (Figure 4) as requested below in response to point 6.

5. Figure 3: The authors should plot % CD25 in B and D and correlate in E % CD25 with % IFN-g (or alternatively MFI CD25 and MFI IFNg).

We have revised Figure 3 as requested. The overall conclusion of the data does not change although, in fact, the statistical significance of some of the comparisons has increased. We have amended the text on pages 5 and 6 to reflect this.

6. Figure 4: Again it would be nice to see the FACS plots of IFN-g production and whether or not IFN-g-producing cells are enriched in the CD25+ cells.

We have modified Figure 4A to show CD25 vs IFN-γ expression in individual cells. As a result, we have also slightly modified the text on page 7.

7. It is unclear, based on the weak ex vivo IFN staining in NK cells, why the authors have not blocked in vivo or a least during the harvesting of the cells cytokine secretion by BFA - or have they done it and not mentioned it?

We have not attempted to block cytokine secretion with BFA in vivo. We know that this has been done in some studies, (e,g, Liu and Whitton J. Immunol, 2005), but it requires intravenous injection of BFA dissolved in DMSO and controls would need to receive i-v DMSO alone. DMSO is extremely toxic and in order to obtain Home Office approval for its use in vivo we would have needed a very good rationale. We do not believe that we have a rationale for such a toxic intervention.

In the study by Liu and Whitton, the purpose was to look at antigen specific T cells, which are present at extremely low frequency, over a time course of up to 3 weeks after virus infection. They were interested in this approach as they were not able to detect any cytokine producing T cells ex vivo and had to resort to in

vitro restimulation to see a response. This is a very different scenario to our experiment where a response was clearly detectable ex vivo. BFA might have enhanced the overall % of positive cells but is unlikely to have changed the interpretation of the experiment.

We did not add BFA to the cells during harvesting as all samples for ex vivo analysis were processed and fixed within 2 hours of collection and were kept on ice and centrifuged at 4oC. This information has been added to the Methods (page 13).

Fourth Editorial Decision – 3 September 2015

Dear Dr. Riley, dear Dr. Stegmann,

It is a pleasure to provisionally accept your manuscript entitled "IL-18 induces expression of the high affinity IL-2 receptor on murine NK cells and is essential for NK cell IFN-γ production during murine Plasmodium yoelii infection" for publication in the European Journal of Immunology. For final acceptance, please follow the instructions below and return the requested items as soon as possible as we cannot process your manuscript further until all items listed below are dealt with.

Please note that EJI articles are now published online a few days after final acceptance (see Accepted Articles: http://onlinelibrary.wiley.com/journal/10.1002/(ISSN)1521-4141/accepted). The files used for the Accepted Articles are the final files and information supplied by you in Manuscript Central. You should therefore check that all the information (including author names) is correct as changes will NOT be permitted until the proofs stage.

We look forward to hearing from you and thank you for submitting your manuscript to the European Journal of Immunology.

Yours sincerely,
Karen Chu

on behalf of Prof. Annette Oxenius

Dr. Karen Chu
Editorial Office
European Journal of Immunology
e-mail: ejied@wiley.com
www.eji-journal.eu
